# Molecular subtypes of breast cancer identified by dynamically enhanced MRI radiomics: the delayed phase cannot be ignored

**DOI:** 10.1186/s13244-024-01713-9

**Published:** 2024-05-31

**Authors:** Guoliang Huang, Siyao Du, Si Gao, Liangcun Guo, Ruimeng Zhao, Xiaoqian Bian, Lizhi Xie, Lina Zhang

**Affiliations:** 1 Department of Radiology, The First Hospital of China Medical University, Shenyang, 110001 China; 2 Department of Radiology, Daping Hospital, Army Medical University, Chongqing, 400010 China; 3 GE Healthcare, Beijing, 100176 China; 4 Department of Radiology, The Fourth Hospital of China Medical University, Shenyang, 110165 Liaoning Province China

**Keywords:** Breast neoplasms, Magnetic resonance imaging, Machine learning

## Abstract

**Objectives:**

To compare the diagnostic performance of intratumoral and peritumoral features from different contrast phases of breast dynamic contrast-enhanced magnetic resonance imaging (DCE-MRI) by building radiomics models for differentiating molecular subtypes of breast cancer.

**Methods:**

This retrospective study included 377 patients with pathologically confirmed breast cancer. Patients were divided into training set (*n* = 202), validation set (*n* = 87) and test set (*n* = 88). The intratumoral volume of interest (VOI) and peritumoral VOI were delineated on primary breast cancers at three different DCE-MRI contrast phases: early, peak, and delayed. Radiomics features were extracted from each phase. After feature standardization, the training set was filtered by variance analysis, correlation analysis, and least absolute shrinkage and selection (LASSO). Using the extracted features, a logistic regression model based on each tumor subtype (Luminal A, Luminal B, HER2-enriched, triple-negative) was established. Ten models based on intratumoral or/plus peritumoral features from three different phases were developed for each differentiation.

**Results:**

Radiomics features extracted from delayed phase DCE-MRI demonstrated dominant diagnostic performance over features from other phases. However, the differences were not statistically significant. In the full fusion model for differentiating different molecular subtypes, the most frequently screened features were those from the delayed phase. According to the Shapley additive explanation (SHAP) method, the most important features were also identified from the delayed phase.

**Conclusions:**

The intratumoral and peritumoral radiomics features from the delayed phase of DCE-MRI can provide additional information for preoperative molecular typing. The delayed phase of DCE-MRI cannot be ignored.

**Critical relevance statement:**

Radiomics features extracted and radiomics models constructed from the delayed phase of DCE-MRI played a crucial role in molecular subtype classification, although no significant difference was observed in the test cohort.

**Key Points:**

- The molecular subtype of breast cancer provides a basis for setting treatment strategy and prognosis.
- The delayed-phase radiomics model outperformed that of early-/peak-phases, but no differently than other phases or combinations.
- Both intra- and peritumoral radiomics features offer valuable insights for molecular typing.

**Graphical Abstract:**

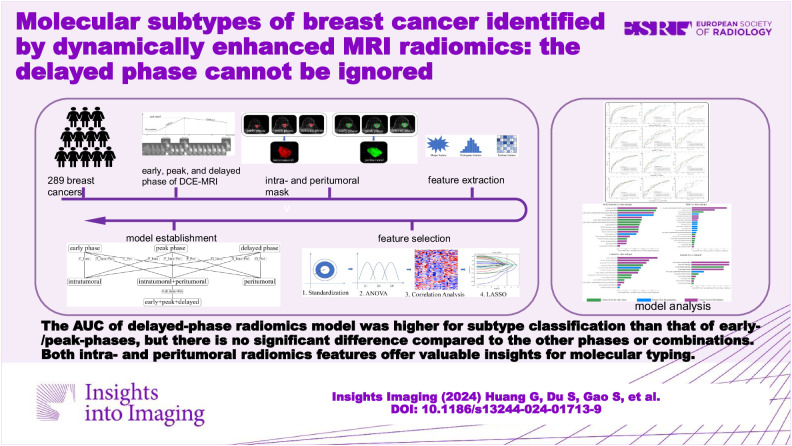

## Background

Breast cancer is the most frequently occurring malignancy in women [1], with complex biological behaviors and different molecular subtypes. The classification of molecular subtypes provides a basis for setting the treatment strategy and prognosis for breast cancer patients. Currently, preoperative determination of molecular subtypes relies on immunohistochemical analysis of biopsy samples, but small specimens may not fully represent the entire tumor. Therefore, there is a need for a non-invasive and reliable diagnostic method that can provide a comprehensive assessment of the tumor’s pathological status to aid in treatment decisions.

Dynamic contrast-enhanced magnetic resonance imaging (DCE-MRI) is useful to quantitatively assess the permeability and perfusion of subtle microvascular environment. Radiomics involves high-throughput computing to extract many quantitative features from medical imaging, allowing the prediction of the tumor phenotype through mathematic models built with selected radiomics features [2]. Prior studies have reported that DCE-MRI based texture analysis can be used to detect Ki-67 [3, 4] and human epidermal growth factor receptor 2 (HER2) status [3], determine molecular subtypes [5, 6], identify sentinel lymph node metastasis [7, 8], and evaluate the response to neoadjuvant chemotherapy [9, 10]. However, these studies only extracted intratumoral features from the first phase [4, 5, 7, 11] or the peak phase [3, 12] following enhancements. Due to the variability of different scan machines and technologies, no consensus has been reached on which phase could offer the best performance for the molecular subtype determination.

Tumor cell-induced peritumoral microenvironment is involved in tumor spread and development [13, 14], with peritumoral cells exhibiting a more critical role than intratumoral cells in the clinical outcome of breast cancer [15]. Previous studies have found that molecular subtypes of cancer distinguished by radiomics analysis, extracting peritumoral features, exhibit comparability and complementarity compared to intratumoral features [3]. However, the extraction of these peritumoral features always accompanies the extraction of intratumoral features, which is based on the early phase [16] or peak phase [3]. It is also crucial to comprehensively evaluate the value of peritumoral features based on different phases of DCE-MRI.

This study extracted comprehensive intratumoral and peritumoral features under different DCE-MRI phases. Multiple radiomics models were established and compared using two regions (intratumoral and peritumoral) and three phases (early, peak, and delayed). The study aims to disclose the most favorable phase and radiomics features for molecular subtype differentiation.

## Materials and methods

### Study population

**Fig. 1:**
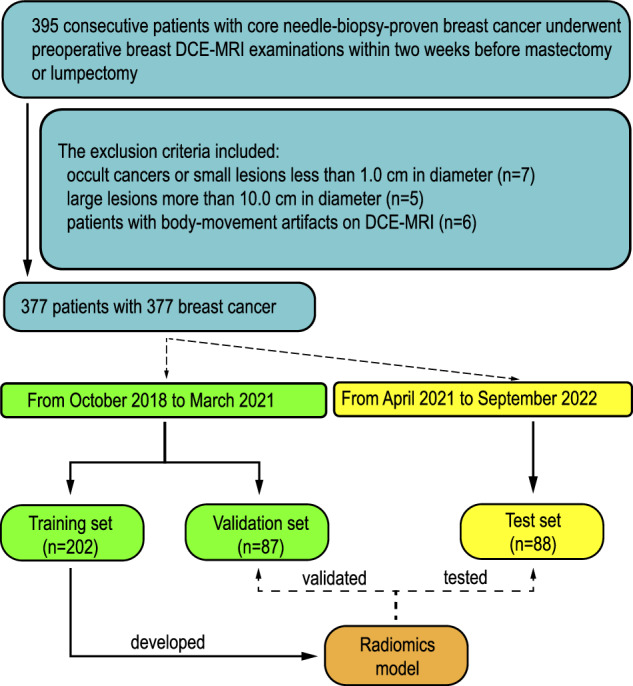
The flowchart of patients’ selection

The hospital ethics committees approved the design of this study. Due to the study’s retrospective nature, the requirement for patient approval or written informed consent was waived. From October 2018 to September 2022, 395 consecutive patients with core needle-biopsy-proven breast cancer underwent preoperative breast DCE-MRI examinations within two weeks before mastectomy or lumpectomy in our hospital. The exclusion criteria included: (1) occult cancers or small lesions less than 1.0 cm in diameter (*n* = 7); (2) large lesions more than 10.0 cm in diameter (*n* = 5); (3) patients with body-movement artifacts on DCE-MRI (*n* = 6). Only the largest lesion was selected for analysis for patients with multiple lesions in the unilateral breast. Patients from October 2018 and March 2021 were included as training set (*n* = 202) and validation set (*n* = 87). From April 2021 to September 2022, the eligible patients were included in the test set (*n* = 88). The flowchart of patients’ selection and process of dataset construction are shown in Fig. 1. This dataset was not investigated in our previous paper, and no public datasets were used in the study. The CLEAR checklist was used to guide reporting (see the checklist in Supplementary Table S1).

### Acquisition of clinicopathological features

Clinical information for each patient included age, lesion size, menstrual status, histology, lymph node metastasis, and histological grade. Estrogen receptor (ER), progesterone receptor (PR), and HER2 were evaluated according to the ASCO/USCAP guidelines [17, 18] using an avidin-biotin immunohistochemistry technique for biopsy specimens. Additionally, Ki-67 expression was classified as high if the staining positivity was more than 20% and low if it was equal to or less than 20% [19]. The molecular subtypes of breast cancer were divided into (1) Luminal: ER+ and/or PR+, which includes Luminal A (ER+ and/or PR+, HER2− and low Ki-67 expression) and Luminal B (ER+ and/or PR+ and HER2+ or high Ki-67 expression); (2) HER2-enriched: ER− and PR−, HER2+; (3) TNBC: ER−, PR−, HER2−.

### MRI acquisition

Breast MRI examinations were performed using a a 3-T MR scanner (SIGNATM Pioneer, GE Healthcare, Milwaukee, WI, USA) in the prone position with an 8-channel breast coil. After conventional sequences, including T1WI, T2WI, and diffusion-weighted imaging (DWI), differential subsampling with cartesian ordering (DISCO) technology with fat suppression was used to obtain the T1-weighted DCE-MRI sequence. Following the first pre-contrast scanning followed by a pause of 20 s, the contrast agent (Gadodiamine, GE Healthcare) was injected intravenously as a bolus (0.1 mmol/kg) followed by flushing with 20 mL of saline, both at a rate of 2 mL/s. A total of 18 phases (temporal resolution = 19.4 s/phase) were subsequently acquired without interruption. Scanning parameters are reported in Supplementary Table S2.

**Fig. 2:**
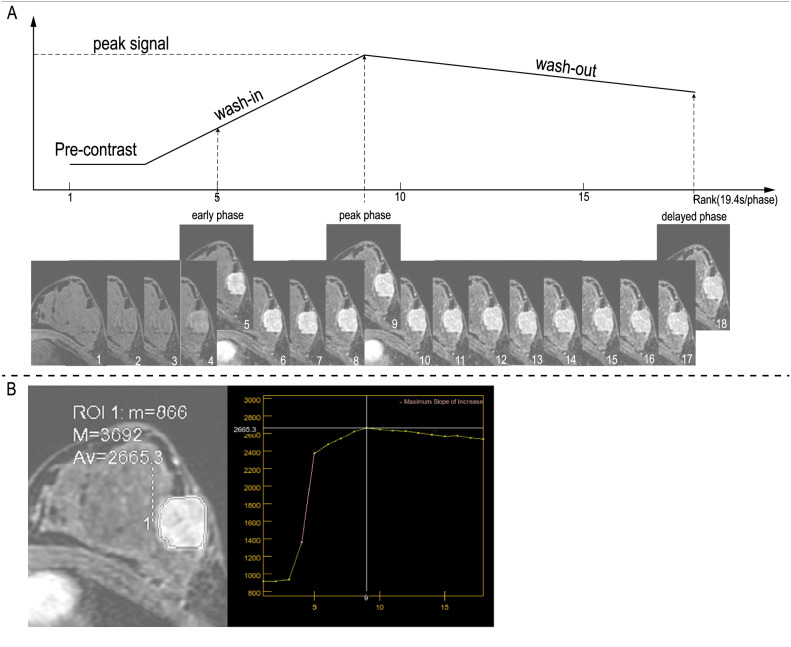
The process of DCE-MRI used in this study. Figure 2A A total of 18 phases of DCE-MRI was scanned in this study, but images from three phases were selected for feature extraction: the early phase (5th post-contrast phase), peak phase (9th post-contrast phase), and delayed phase (18th post-contrast phase). Figure 2B A 22-year-old woman with Luminal B breast cancer confirmed by pathological immunohistochemistry. Shown is the time-signal intensity curve for this patient

All images in this study were downloaded from our institutional picture archiving and communication system. Images from three phases were collected for analysis, including the early phase (5th post-contrast phase), peak phase (9th post-contrast phase), and delayed phase (18th post-contrast phase), as demonstrated in Fig. 2.

### Tumor segmentation and feature extraction

**Fig. 3:**
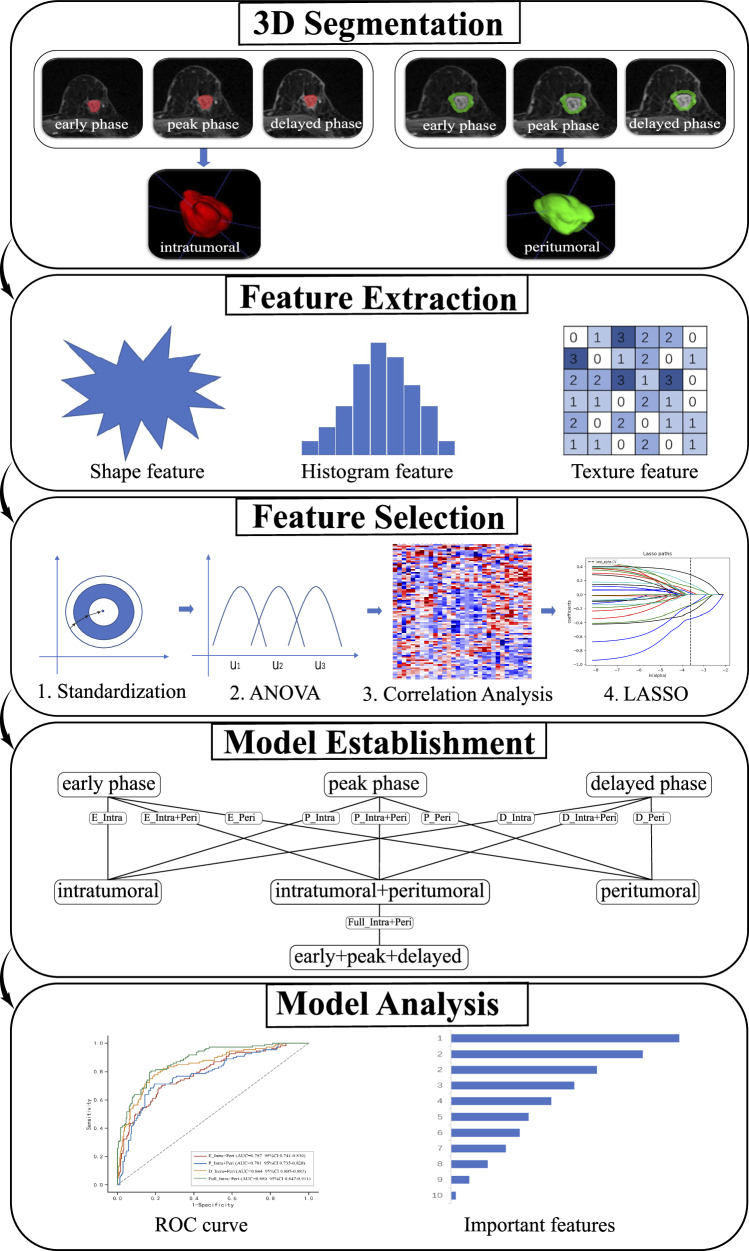
Workflow of the radiomics analysis

DCE-MRI data from all patients were converted from DICOM into NIFTI format for tumor segmentation using ITK-SNAP software (http://www.itksnap.org/) and for feature extraction using Artificial Intelligent Kit (A.K.) software (GE Healthcare, Shanghai, Version 3.0.1). The processing workflow encompasses three sequential steps (Fig. 3).

Step 1. Intratumoral volume of interest (VOI) delineation. Each tumor VOI on the peak phase of DCE-MRI was created by threshold-based, seed point driven, semi-automatic segmentation using ITK-SNAP [20]. Radiologist A with five years’ experience in breast imaging performed VOI delineation on the peak phase of DCE-MRI, then copied to the early and delayed phase images and performed manual adjustment. Radiologist B with ten years’ experience in breast imaging independently segmented tumor masks on 40 randomly selected samples using the same method. Intraclass correlation coefficient (ICC) was utilized for evaluating the interobserver agreement in terms of VOI-based radiomics features extraction. An ICC value of 0.75 or greater was considered an excellent interobserver reproducibility and was retained. To fully mask the tumor heterogeneity, cystic and necrotic areas of the tumor were included.

Step 2. Peritumoral VOI delineation. Annular intratumoral regions (not containing the lesion area) of 5 mm were obtained automatically by dilating the delineated lesion contours using A.K. software. A morphologic dilation operation was also performed to capture the perilesional region [21]. If the contours from intratumoral areas were beyond the parenchyma of the breast after expansion, the portion beyond the parenchyma was manually removed. Interobserver agreement of peritumoral features of each phase was also assessed.

Step 3. Feature extraction. 106 intratumoral and 106 peritumoral radiomics features were extracted from each DCE-MRI phase. These features were defined by PyRadiomics (http://pyradiomics.readthedocs.io/en/latest/index.html), including 14 shape features, 17 first order (FO) features, 24 Gray Level Co-occurrence Matrix (GLCM) features, 14 Gray Level Dependence Matrix (GLDM) features, 16 Gray Level Run Length Matrix (GLRLM) features, 16 Gray Level Size Area Matrix Gray Level Size Zone Matrix (GLSZM) features, 5 Neighboring Gray Tone Difference Matrix (NGTDM) features.

### Feature selection

We used the stratified random sampling method to divide development set into a training and a validation set at a 7:3 ratio. The synthetic minority oversampling technique (SMOTE) was used in the training set to overcome data imbalance in the training set, and oversampled the number of patients with HER2-enriched and TNBC to twice their own. The same step-by-step process was used for feature standardization, feature selection, and model construction in the training set for each model.

Feature selection was conducted in the following steps: (1) *Z*-Score feature standardization using the following equation: Standardized value = (original value − average value) / standard deviation; (2) variance analysis to eliminate features with variance less than 1; (3) correlation analysis to retain one feature when the correlation coefficient between multiple features was greater than 0.7; (4) the least absolute shrinkage and selection operator (LASSO) method with 5-fold cross-validation was used to select the optimal subset of distinguishable features.

### Model establishment and comparisons

Logistic regression models were built to distinguish one subtype from the others (Luminal vs. the others, HER2-enriched vs. the others, TNBC vs. the others). The Luminal subtype was further distinguished as the Luminal A and Luminal B subtype. For each differentiation, we established six radiomics models for the one-to-one analysis of phase and region, three combined models using intratumoral plus peritumoral features, and one full fusion model. Models for single phase analysis included: the intratumoral region from the early phase (E_Intra), the peak phase (P_Intra), the delayed phase (D_Intra); the peritumoral region from the early phase (E_Peri), the peak phase (P_Peri), the delayed phase (D_Peri). Models for combined analysis included: the intratumoral and peritumoral region from the early phase (E_Intra+Peri), the peak phase (P_Intra+Peri), the delayed phase (D_Intra+Peri). The full fusion model was the intratumoral and peritumoral region from full phases (Full_Intra+Peri). The test data only be used once for evaluation of the final model to prevent optimistic biases.

The diagnostic abilities of the training and validation set were evaluated by receiver operator characteristics (ROC) curves. The Shapley additive explanation (SHAP) method was utilized to explain the models by evaluating the contribution of each feature to the differentiation.

### Statistical analysis

All statistical analyses, feature selection, and model building in this study were performed using R 3.5.1 and Python 3.5.6. Continuous variables are described by median (interquartile range), and categorical variables are described by frequency. Continuous variables were compared using a Mann–Whitney *U* test or the Kruskal–Wallis test. For categorical variables, Chi-square and Fisher exact tests were performed appropriately. The AUC values of different models were compared using the Delong test. A *p*-value < 0.05 was considered statistically significant. SHAP values were computed with the Python package SHAP (https://github.com/slundberg/shap).

## Results

### Patient characteristics

The molecular subtype of the 377 patients included in the study was as follows: Luminal (*n* = 224, Luminal A *n* = 73 and Luminal B *n* = 151), HER2-enriched (*n* = 78), and TNBC (*n* = 75). The detailed characteristics of the patients are summarized in Table 1. Tumor size (*p* < 0.001), lymph node metastasis (*p* < 0.001), histological type (*p* = 0.012) and grade (*p* < 0.001) significantly differed among the Luminal, HER2-enriched and TNBC subtypes. No significant differences were observed in term of age (*p* = 0.487), menopausal status (*p* = 0.381).

**Table 1:** Clinical and pathological characteristics

Characteristics	All (*n* = 377)	Luminal A (*n* = 73)	Luminal B (*n* = 151)	Her2-enriched (*n* = 78)	TNBC (*n* = 75)	*p*-value
Age, years	52 (44, 58)	52 (42, 59)	51 (44, 57)	52 (44, 57)	53 (45, 60)	0.487
Size, mm	28 (20, 40)	20 (15, 29)	28 (20, 42)	33 (24, 46)	30 (23, 42)	< 0.001
Menopausal						0.381
Premenopausal	183 (48.54)	37 (50.68)	72 (47.68)	40 (51.28)	45 (60.00)	
Postmenopausal	194 (51.46)	36 (49.32)	79 (52.32)	38 (48.72)	30 (40.00)	
ER						< 0.001
Negative	151 (40.05)	0 (0.00)	3 (1.99)	75 (96.15)	73 (97.33)	
Positive	226 (59.95)	73 (100.00)	148 (98.01)	3 (3.85)	2 (2.67)	
PR						< 0.001
Negative	145 (38.46)	1 (1.37)	12 (7.95)	67 (85.90)	65 (86.67)	
Positive	232 (61.54)	72 (98.63)	139 (92.05)	11 (14.10)	10 (13.33)	
HER-2						< 0.001
Negative	233 (61.80)	73 (100.00)	85 (56.29)	0 (0.00)	75 (100.00)	
Positive	144 (38.20)	0 (0.00)	66 (43.71)	78 (100.00)	0 (0.00)	
Ki67 status						< 0.001
≤ 20%	99 (26.26)	73 (100.00)	12 (7.95)	10 (12.82)	4 (5.33)	
> 20%	278 (73.74)	0 (0.00)	139 (92.05)	68 (87.18)	71 (94.67)	
Histology						0.012
IDC	314 (83.29)	53 (72.60)	129 (85.43)	63 (80.77)	69 (92.00)	
Others	63 (16.71)	20 (27.40)	22 (14.57)	15 (19.23)	6 (8.00)	
LN metastasis						< 0.001
Negative	176 (46.68)	51 (69.86)	56 (37.09)	44 (56.41)	25 (33.33)	
Positive	201 (53.32)	22 (30.14)	95 (62.91)	34 (43.59)	50 (66.67)	
Histological grade						< 0.001
I	19 (5.04)	11 (15.07)	3 (1.99)	3 (3.85)	2 (2.67)	
II	251 (66.58)	59 (80.82)	112 (74.17)	50 (64.10)	30 (40.00)	
III	107 (28.38)	3 (4.11)	36 (23.84)	25 (32.05)	43 (57.33)	

Continuous variables are described by median (interquartile range); categorical variables are described by numbers (percentages)

*ER* estrogen receptor, *PR* progesterone receptor, *HER2* human epidermal growth factor receptor 2, *TNBC* triple-negative breast cancer, *IDC* invasive ductal carcinoma, *LN* lymph node

### Repeatability analysis

Excellent interobserver agreement in tumor masking and radiomics feature extraction was attained, with an ICC value exceeding 0.90 for intratumoral and peritumoral features on different phases of DCE-MRI between the masks generated by the two radiologists.

### Analysis of phase and region one-to-one model

Selected features of each model can be found in Supplementary Tables S3–S6. In the test set, the D_Intra model had the highest performance (AUC = 0.735, 95% CI: 0.606–0.864) in differentiating the HER2-enriched subtype, with a sensitivity of 0.654, specificity of 0.845, and accuracy of 0.796. The D_Intra model also had the highest performance in differentiating the Luminal subtype (AUC = 0.712, 95% CI: 0.603–0.821), TNBC (AUC = 0.668, 95% CI: 0.536–0.800) from the others, and distinguishing between Luminal A and Luminal B within the Luminal subtype (AUC = 0.682, 95% CI: 0.458–0.863). However, the comparison of ROC curves verified by the DeLong test showed no statistical difference. The AUCs of various models in the training set, validation set and test set are shown in Tables 2–4.

**Table 2:** Performance of differentiating HER2-enriched using phase and region one-to-one model

Model	Training				Validation				Test			
	AUC (95% CI)	SEN	SPE	ACC	AUC (95% CI)	SEN	SPE	ACC	AUC (95% CI)	SEN	SPE	ACC
E_Intra	0.713 (0.662, 0.764)	0.500	0.852	0.744	0.632 (0.507, 0.752)	0.312	0.831	0.736	0.666 (0.526, 0.807)	0.539	0.871	0.773
E_Peri	0.755 (0.708, 0.799)	0.713	0.697	0.702	0.731 (0.624, 0.834)	0.625	0.718	0.701	0.694 (0.557, 0.830)	0.506	0.897	0.784
P_Intra	0.666 (0.617, 0.714)	0.815	0.529	0.616	0.612 (0.471, 0.744)	0.688	0.521	0.552	0.617 (0.460, 0.774)	0.423	0.899	0.807
P_Peri	0.786 (0.741, 0.828)	0.796	0.693	0.724	0.728 (0.587, 0.855)	0.625	0.775	0.747	0.696 (0.572, 0.820)	0.498	0.855	0.750
D_Intra	0.763 (0.717, 0.807)	0.676	0.738	0.719	0.740 (0.625, 0.846)	0.562	0.775	0.736	0.735 (0.606, 0.864)	0.654	0.845	0.796
D_Peri	0.718 (0.668, 0.765)	0.593	0.783	0.724	0.716 (0.603, 0.821)	0.375	0.845	0.759	0.690 (0.561, 0.820)	0.519	0.919	0.796

No statistically significant difference was observed (Delong test)

*E_Intra* intratumoral region from the early phase, *E_Peri* peritumoral region from the early phase, *P_Intra* intratumoral region from the peak phase, *P_Peri* peritumoral region from the peak phase, *D_Intra* intratumoral region from the delayed phase, *D_Peri* peritumoral region from the delayed phase, *AUC* area under the ROC curve, *CI* confidence interval, *SEN* sensitivity, *SPE* specificity, *ACC* accuracy

**Table 3:** Performance of differentiating TNBC using phase and region one-to-one model

Model	Training				Validation				Test			
	AUC (95% CI)	SEN	SPE	ACC	AUC (95% CI)	SEN	SPE	ACC	AUC (95% CI)	SEN	SPE	ACC
E_Intra	0.682 (0.634, 0.730)	0.803	0.515	0.611	0.656 (0.539, 0.763)	0.833	0.536	0.598	0.657 (0.530, 0.785)	0.893	0.429	0.534
E_Peri	0.695 (0.645, 0.740)	0.692	0.617	0.642	0.563 (0.423, 0.699)	0.500	0.522	0.517	0.606 (0.467, 0.745)	0.833	0.386	0.477
P_Intra	0.623 (0.573, 0.671)	0.692	0.519	0.577	0.523 (0.409, 0.644)	0.611	0.522	0.540	0.681 (0.454, 0.783)	0.523	0.814	0.750
P_Peri	0.651 (0.602, 0.696)	0.966	0.315	0.531	0.610 (0.474, 0.737)	0.833	0.232	0.356	0.632 (0.470, 0.794)	0.510	0.829	0.761
D_Intra	0.763 (0.718, 0.806)	0.744	0.694	0.710	0.694 (0.574, 0.811)	0.611	0.623	0.621	0.668 (0.536, 0.800)	0.561	0.743	0.705
D_Peri	0.660 (0.611, 0.708)	0.615	0.651	0.639	0.630 (0.500, 0.752)	0.556	0.609	0.598	0.640 (0.495, 0.784)	0.556	0.686	0.659

No statistically significant difference was observed (Delong test)

*E_Intra* intratumoral region from the early phase, *E_Peri* peritumoral region from the early phase, *P_Intra* intratumoral region from the peak phase, *P_Peri* peritumoral region from the peak phase, *D_Intra* intratumoral region from the delayed phase, *D_Peri* peritumoral region from the delayed phase, *AUC* area under the ROC curve, *CI* confidence interval, *SEN* sensitivity, *SPE* specificity, *ACC* accuracy

**Table 4:** Performance of differentiating Luminal form the others and Luminal A form Luminal B within the Luminal using phase and region one-to-one model

Model	Training				Validation				Test			
	AUC (95% CI)	SEN	SPE	ACC	AUC (95% CI)	SEN	SPE	ACC	AUC (95% CI)	SEN	SPE	ACC
Luminal vs. Other												
E_Intra	0.682 (0.632, 0.73)	0.732	0.578	0.634	0.681 (0.580, 0.773)	0.755	0.500	0.655	0.672 (0.559, 0.785)	0.648	0.500	0.796
E_Peri	0.688 (0.639, 0.737)	0.488	0.822	0.702	0.684 (0.577, 0.784)	0.585	0.676	0.621	0.681 (0.566, 0.797)	0.773	0.614	0.693
P_Intra	0.643 (0.588, 0.693)	0.748	0.471	0.571	0.549 (0.440, 0.655)	0.660	0.412	0.563	0.703 (0.594, 0.812)	0.636	0.727	0.682
P_Peri	0.694 (0.645, 0.742)	0.717	0.609	0.648	0.669 (0.562, 0.768)	0.868	0.412	0.690	0.676 (0.560, 0.792)	0.705	0.682	0.693
D_Intra	0.726 (0.678, 0.772)	0.748	0.609	0.659	0.714 (0.621, 0.806)	0.736	0.588	0.678	0.712 (0.603, 0.821)	0.614	0.773	0.693
D_Peri	0.689 (0.640, 0.736)	0.567	0.707	0.656	0.674 (0.576, 0.765)	0.660	0.500	0.598	0.697 (0.583, 0.810)	0.773	0.636	0.705
Luminal A vs. Luminal B												
E_Intra	0.621 (0.508, 0.734)	0.641	0.591	0.599	0.583 (0.423, 0.744)	0.718	0.688	0.562	0.615 (0.410, 0.819)	0.865	0.264	0.432
E_Peri	0.632 (0.517, 0.746)	0.640	0.631	0.625	0.601 (0.450, 0.752)	0.882	0.405	0.537	0.629 (0.411, 0.848)	0.520	0.824	0.750
P_Intra	0.570 (0.451, 0.690)	0.490	0.694	0.617	0.565 (0.403, 0.728)	0.636	0.563	0.574	0.522 (0.376, 0.768)	0.489	0.794	0.705
P_Peri	0.686 (0.577, 0.796)	0.563	0.784	0.692	0.569 (0.406, 0.732)	0.688	0.553	0.500	0.582 (0.407, 0.758)	0.896	0.441	0.546
D_Intra	0.711 (0.606, 0.815)	0.592	0.775	0.699	0.637 (0.450, 0.881)	0.778	0.638	0.668	0.682 (0.458, 0.863)	0.469	0.871	0.841
D_Peri	0.658 (0.550, 0.767)	0.685	0.627	0.639	0.620 (0.452, 0.788)	0.625	0.684	0.611	0.635 (0.427, 0.843)	0.386	0.892	0.796

No statistically significant difference was observed (Delong test)

*E_Intra* intratumoral region from the early phase, *E_Peri* peritumoral region from the early phase, *P_Intra* intratumoral region from the peak phase, *P_Peri* peritumoral region from the peak phase, *D_Intra* intratumoral region from the delayed phase, *D_Peri* peritumoral region from the delayed phase, *AUC* area under the ROC curve, *CI* confidence interval, *SEN* sensitivity, *SPE* specificity, *ACC* accuracy

### Analysis of intra- plus peri-tumoral combination model

**Fig. 4:**
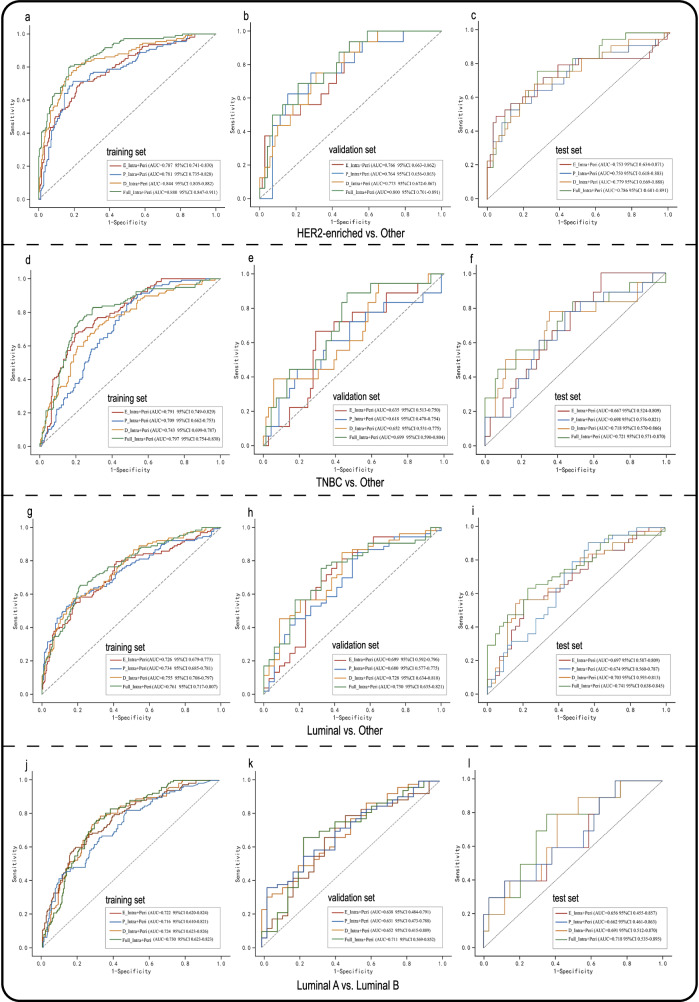
Receiver operating characteristic (ROC) curves of combined model and full fusion model. E_Intra + Peri, intratumoral and peritumoral region from the early phase; P_Intra + Peri, intratumoral and peritumoral region from the peak phase; D_Intra + Peri, intratumoral and peritumoral region from the delayed phase; Full_Intra + Peri, intratumoral and peritumoral region from full phases

Tables S7–S10 presented the selected features of each intra- plus peri-tumoral combination model. The AUCs of combined intratumoral and peritumoral features from the delayed-phase model (D_Intra+Peri) were higher than the early-phase and peak-phase model in training, validation and test set. In the test set, the AUCs were 0.779 (95% CI: 0.669–0.888) for HER2-enriched, 0.703 (95% CI: 0.593–0.813) for Luminal subtype, 0.718 (95% CI: 0.570–0.866) for TNBC differentiation from the others, and 0.691 (95% CI 0.512–0.870) for Luminal A and Luminal B differentiation using D_Intra+Peri model. D_Intra+Peri model had similar diagnostic performance with Full_Intra+Peri model (AUC = 0.786, 0.721, 0.741, and 0.718, respectively) and no significant statistical difference was observed in the validation set (all Delong *p* > 0.05, Table 5). Figure 4 shows the ROC curves in the training set, validation set and test set.

**Table 5:** Comparison of prediction performance on the validation set of combined image feature model

Model	Training AUC (95% CI)	*p* value*	Validation AUC (95% CI)	*p* value*	Test AUC (95% CI)	*p* value*
HER2-enriched vs. Other						
E_Intra + Peri	0.787 (0.741, 0.830)	**< 0.001**	0.766 (0.663, 0.862)	0.396	0.753 (0.634, 0.871)	0.614
P_Intra + Peri	0.781 (0.735, 0.828)	**< 0.001**	0.764 (0.656, 0.863)	0.466	0.750 (0.618, 0.883)	0.601
D_Intra + Peri	0.844 (0.805, 0.882)	**0.041**	0.773 (0.672, 0.867)	0.599	0.779 (0.669, 0.888)	0.115
Full_Intra + Peri	0.880 (0.847, 0.911)	…	0.800 (0.701, 0.891)	…	0.786 (0.681, 0.891)	…
TNBC vs. Other						
E_Intra + Peri	0.791 (0.749, 0.829)	0.833	0.635 (0.513, 0.750)	0.274	0.667 (0.524, 0.809)	0.578
P_Intra + Peri	0.709 (0.662, 0.753)	**0.004**	0.618 (0.478, 0.754)	0.241	0.698 (0.576, 0.821)	0.735
D_Intra + Peri	0.743 (0.699, 0.787)	**0.034**	0.652 (0.531, 0.775)	0.309	0.718 (0.570, 0.866)	0.784
Full_Intra + Peri	0.797 (0.754, 0.838)	…	0.699 (0.590, 0.804)	…	0.721 (0.571, 0.870)	…
Luminal vs. Other						
E_Intra + Peri	0.726 (0.679, 0.773)	0.194	0.699 (0.592, 0.796)	0.528	0.697 (0.587, 0.809)	0.512
P_Intra + Peri	0.734 (0.685, 0.781)	0.428	0.680 (0.577, 0.775)	0.436	0.674 (0.560, 0.787)	0.339
D_Intra + Peri	0.755 (0.708, 0.797)	0.794	0.728 (0.634, 0.818)	0.965	0.703 (0.593, 0.813)	0.206
Full_Intra + Peri	0.761 (0.717, 0.807)	…	0.730 (0.635, 0.821)	…	0.741 (0.638, 0.845)	…
Luminal A vs. Luminal B						
E_Intra + Peri	0.722 (0.620, 0.824)	0.888	0.638 (0.484, 0.791)	0.477	0.656 (0.455, 0.857)	0.366
P_Intra + Peri	0.716 (0.610, 0.821)	0.238	0.631 (0.473, 0.788)	0.562	0.662 (0.461, 0.863)	0.408
D_Intra + Peri	0.724 (0.623, 0.826)	0.956	0.652 (0.415, 0.889)	0.917	0.691 (0.512, 0.870)	0.745
Full_Intra + Peri	0.730 (0.623, 0.823)	…	0.711 (0.569, 0.852)	…	0.718 (0.535, 0.895)	…

*E_Intra + Peri* intratumoral and peritumoral region from the early phase, *P_Intra + Peri* intratumoral and peritumoral region from the peak phase, *D_Intra + Peri* intratumoral and peritumoral region from the delayed phase, *Full_Intra + Peri* intratumoral and peritumoral region from full phases, *HER2* human epidermal growth factor receptor-2, *TNBC* triple-negative breast cancer, *AUC* area under the ROC curve, *CI* confidence interval

* The delong test was used to compare with the Full_Intra + Peri model, and a *p* < 0.05 was considered statistically significant

Statistically significant *p* < 0.05 values are in bold

### Analysis of the full fusion model

The selected features of the full fusion model (Full_Intra+Peri model) are provided in Table S11. The AUC value of Full_Intra+Peri model was the highest among all models for HER2-enriched, TNBC and Luminal subtype differentiating from the others and Luminal A and Luminal B differentiation within the Luminal subtype (Table 5).

**Fig. 5:**
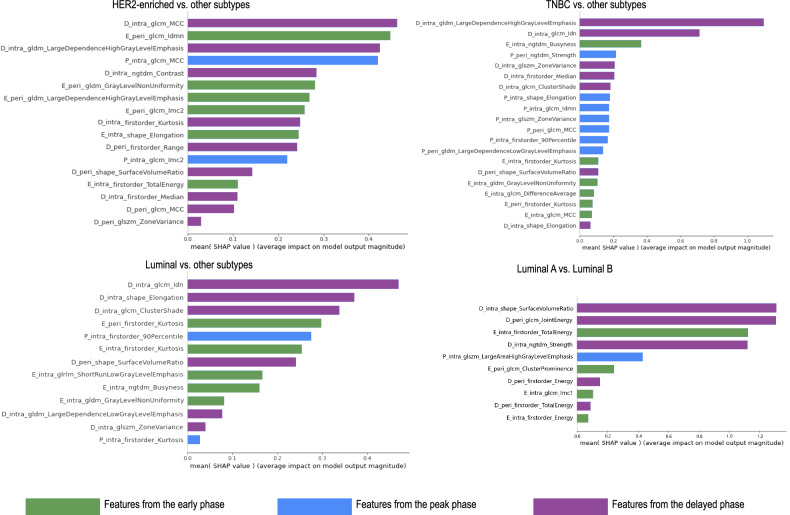
The selected feature sets of the Full_Intra + Peri model were evaluated through the shapley additive explanations (SHAP). The features are listed in descending order according to their contribution to the predict of the molecular subtype of breast cancer

A total of 17 features were selected in the Full_Intra+Peri model for differentiating the HER2-enriched subtype, with most features coming from the delayed phase (9/17). Among the top five features with the highest SHAP value, three features came from the delayed phase, and the most important feature was D_Intra_glcm_MCC (Fig. 5). Likewise, features from the delayed phase predominated in the models of TNBC (7/20), Luminal (6/13) differentiation from the others and Luminal A and Luminal B (5/10) differentiation within the Luminal subtype. In these models, the most important features were identified from the delayed phase and most of the top five important features came from the delayed phase.

## Discussion

In this work, we built and compared multiple models for differentiating molecular subtypes by extracting intra- and peri-tumoral features from multiple phases of DCE-MRI. Radiomics features from the delayed phase were proposed with better performance for HER2-enriched, TNBC, and Luminal subtype differentiation from the others and Luminal A and Luminal B differentiation within the Luminal subtype. The outperformance of the delayed contrast phase was also applicable to the intra- plus peri-tumoral combination model. The comprehensive integration of multi-phase and multi-region DCE-MRI radiomics model demonstrated superior performance in differentiating molecular subtypes, with no statistically significant difference observed when compared to the delayed phase radiomics model. Using the SHAP method, most of the top five features in Full_Intra+Peri model were derived from the delayed phase, while the most critical features also came from the delayed phase. Therefore, this result indicated that the delayed contrast phase of DCE-MRI played a non-negligible role in preoperative molecular typing.

The present study constitutes the first attempt to use multiphase DCE-MRI to distinguish the molecular typing of breast cancer and explore the impact of different contrast enhancement phases. An optimized DCE-MRI sequence with high temporal and spatial resolution accurately captured the contrast agent’s retention state and spatial distribution. Due to technical variables, DCE-MRI in previous studies [4, 5, 12] has a poor temporal resolution (about 60–100 s) and comparable spatial resolution. Thus, the radiomics features in these studies were usually extracted from the first enhancement phase of primary tumors [4, 5] and the phase with the most intense contrast enhancement for malignant characterization [12]. The early phase of DCE-MRI can reliably evaluate the presence of invasive cancer after neoadjuvant chemotherapy. In contrast, the delayed phase often better assessed the size of additional carcinoma in situ [22]. The delayed phase is particularly important for residual tumor extent outline in invasive lobular cancer, non-mass enhancement at MRI, and hormone receptor-positive/HER2–negative tumors [22]. Early enhancement on DCE-MRI manifested tumor vascularization and increased malignancy, which is considered to focus on the nature of tumor cells. In the delayed phase, the contrast agent slowly infiltrated and distributed to interstitial tumor tissue, which tended to show the nature of the tumor matrix [23]. The tumor stroma surrounding cancer cells provides a microenvironment for tumor genesis [14], tumor-stimulating inflammation [24], metabolism [25], metastasis [26], chemoresistance [27], and immune escape mechanisms [28]. The tumor interstitial phenotype varies among different molecular subtypes: TNBC was more frequently observed among tumors of inflammatory and normal-like types, whereas Luminal A was prominent in desmoplastic and sclerotic types [29]. The combination of high-throughput imaging features of delayed DCE-MRI reflecting tumor stroma may quantify the difference between different molecular subtypes. The content difference of tumor-infiltrating lymphocytes (TILs) may be another biological explanation. TNBC and HER2-enriched subtypes often recruit more TILs, while only 6% of Luminal subtypes were detected [30]. A previous study [31] compared the multiphase DCE-MRI radiomics model to evaluate the level of TILs in breast cancer. Their results indicated that radiomics features extracted from delayed phase DCE-MRI could help evaluate TIL levels optimally. Feature extraction from the delayed phase achieved better predictability in immune microenvironment analysis. Given our results, the value of the delayed phase in molecular typing may come from the differences in the tumor microenvironment. However, the definitive mechanism needs to be further clarified.

In addition to quantifying intratumoral heterogeneity, peritumoral areas have received increasing attention in tumor imaging. Notably, the expanded annular peritumoral region is regarded as a substitute for the tumor microenvironment. Likewise, the added peritumoral features provide additional value for the differentiation of benign and malignant lesions [32], breast cancer molecular typing [6], and treatment response prediction [33]. Based on the DCE-MRI pharmacokinetic parameters, Li et al [3] found that intra- and peri-tumoral radiomics models yield similar diagnostic performance in differentiating HER2 and Ki67 status. Alternatively, combining intra- and peri-tumoral features significantly improved the model performance [3, 6]. In our study, the separate peritumoral model based on each contrast phase showed equivalent performance compared with the single intratumoral model, especially for the HER2-enriched subtype. We also observed the superiority of the intra- and peri-tumoral combined models. The full fusion model using two-region plus three-phase obtained better performance but was not statistically significant in the validation set. The accuracy of the delay-phase model was the closest to the full fusion model in identifying each molecular subtype. Despite the additional value of the delayed phase, the diagnostic efficiency of radiomics for molecular typing is still not optimistic. The performance of our models was similar to that of established models in most studies, with no significant breakthrough [3, 6, 34]. This may likely be due to the overlap of the various metrics that define molecular subtypes and the complex relationships among receptors hidden behind the images. Although the delayed phase of DCE-MRI showed no significantly improved performance, they still helped amplify distinction in different molecular subtypes.

The high spatiotemporal resolution of DCE-MRI positions it as the foremost technology for accurately capturing tumor interstitial information in conventional imaging sequences. Tang et al [31] and our study emphasized the importance of delayed DCE-MRI in distinguishing TILs level and molecular typing of breast cancer, respectively. Likewise, our publication [9] suggested early DCE-MRI was more useful in predicting neoadjuvant treatment response. Therefore, the DCE-MRI phase should be selected based on different research purposes in the radiomics analysis. The development of specific standards for different DCE-MRI techniques will also promote its clinical application, such as phase selection criteria, available criteria for temporal and spatial resolution, length of delayed scanning time, etc.

There are several noted limitations in our study. First, the radiomics model in this study was established based on single-center and retrospective data with imbalanced sample size, and prospective multicenter studies are needed to further validate our results. Second, this study only employed DISCO DCE-MRI technology to investigate three distinct contrast phases for radiomics analysis, thereby constraining the reproducibility and generalizability of our findings. Our results should be further validated in the standard-of-care MRI protocols as recommended by European Society of Breast Imaging (EUSOBI) guidelines [35] or in the time-resolved MRI techniques from the other vendors [36]. Finally, the extracted features from the delayed contrast phase of DCE-MRI provided valuable information, but there is no clear biological explanation. It is essential to deepen the biological interpretability of intra- and peri-tumoral radiomics features.

## Conclusion

The better performance of radiomics features and models using the delayed phase of DCE-MRI suggests its additional value for preoperative molecular typing, thus the delayed phase of DCE-MRI cannot be ignored in the differentiation of molecular subtypes in breast cancer.

### Supplementary information

ELECTRONIC SUPPLEMENTARY MATERIAL

## Data Availability

The datasets used and/or analyzed during the current study are available from the corresponding author on reasonable request.

## Abbreviations

ACC: Accuracy
CI: Confidence interval
DCE-MRI: Dynamic contrast-enhanced magnetic resonance imaging
DISCO: Differential subsampling with cartesian ordering
ER: Estrogen receptor
HER2: Human epidermal growth factor receptor 2
ICC: Intraclass correlation coefficient
PR: Progesterone receptor
ROC: Receiver operator characteristics
SEN: Sensitivity
SHAP: Shapley additive explanation
SPE: Specificity
TNBC: Triple-negative breast cancer
VOI: Volume of interest
