# Supplementary material for: Molecular subtypes of breast cancer identified by dynamically enhanced MRI radiomics: the delayed phase cannot be ignored
Source: Insights Imaging. 2024 May 31;15:127. doi: 10.1186/s13244-024-01713-9 (PMC11139827; doi:10.1186/s13244-024-01713-9)
Supplement: Supplementary file 1 — ELECTRONIC SUPPLEMENTARY MATERIAL [file 13244_2024_1713_MOESM1_ESM.pdf]

Molecular subtypes of breast cancer identified by dynamically enhanced MRI radiomics: the delayed phase cannot be ignored

ELECTRONIC SUPPLEMENTARY MATERIAL

Table S1 CLEAR checklist

Section	No.	Item	Yes	No	n/a	Page
Title						
	1	Relevant title, specifying the radiomic methodology	☑	☐	☐	1
Abstract						
	2	Structured summary with relevant information	☑	☐	☐	1
Keywords						
	3	Relevant keywords for radiomics	☑	☐	☐	2
Introduction						
	4	Scientific or clinical background	☑	☐	☐	3
	5	Rationale for using a radiomic approach	☑	☐	☐	3-4
	6	Study objective(s)	☑	☐	☐	4
Method						
Study Design	7	Adherence to guidelines or checklists (e.g., CLEAR checklist)	☑	☐	☐	5
	8	Ethical details (e.g., approval, consent, data protection)	☑	☐	☐	4-5
	9	Sample size calculation	☑	☐	☐	5
	10	Study nature (e.g., retrospective, prospective)	☑	☐	☐	5
	11	Eligibility criteria	☑	☐	☐	5
	12	Flowchart for technical pipeline	☑	☐	☐	Figure 1,3
Data	13	Data source (e.g., private, public)	☑	☐	☐	5
	14	Data overlap	☑	☐	☐	5
	15	Data split methodology	☑	☐	☐	5-8
	16	Imaging protocol (i.e., image acquisition and processing)	☑	☐	☐	6
	17	Definition of non-radiomic predictor variables	☐	☐	☑	
	18	Definition of the reference standard (i.e., outcome variable)	☑	☐	☐	5
Segmentation	19	Segmentation strategy	☑	☐	☐	6-7
	20	Details of operators performing segmentation	☑	☐	☐	6-7
Pre-processing	21	Image pre-processing details	☐	☐	☑	
	22	Resampling method and its parameters	☐	☐	☑	
	23	Discretization method and its parameters	☐	☐	☑	
	24	Image types (e.g., original, filtered, transformed)	☑	☐	☐	6

Feature extraction	25	Feature extraction method	☑	☐	☐	7
	26	Feature classes	☑	☐	☐	7
	27	Number of features	☑	☐	☐	7
	28	Default configuration statement for remaining parameters	☑	☐	☐	7-8
Data preparation	29	Handling of missing data	☐	☐	☑	
	30	Details of class imbalance	☑	☐	☐	7-8
	31	Details of segmentation reliability analysis	☑	☐	☐	7
	32	Feature scaling details (e.g., normalization, standardization)	☑	☐	☐	8
	33	Dimension reduction details	☑	☐	☐	8
Modeling	34	Algorithm details	☑	☐	☐	9
	35	Training and tuning details	☑	☑	☐	
	36	Handling of confounders	☐	☐	☑	
	37	Model selection strategy	☑	☐	☐	8
Evaluation	38	Testing technique (e.g., internal, external)	☑	☐	☐	5
	39	Performance metrics and rationale for choosing	☑	☐	☐	8
	40	Uncertainty evaluation and measures (e.g., confidence intervals)	☑	☐	☐	8
	41	Statistical performance comparison (e.g., DeLong’s test)	☑	☐	☐	9
	42	Comparison with non-radiomic and combined methods	☑	☐	☐	9
	43	Interpretability and explainability methods	☑	☐	☐	Figure 5
Results						
	44	Baseline demographic and clinical characteristics	☑	☐	☐	9
	45	Flowchart for eligibility criteria	☑	☐	☐	Figure 1
	46	Feature statistics (e.g., reproducibility, feature selection)	☑	☐	☐	Supplementary Tables S3-S11
	47	Model performance evaluation	☑	☐	☐	Table 2-5
	48	Comparison with non-radiomic and combined approaches	☐	☐	☑	
Discussion						
	49	Overview of important findings	☑	☐	☐	11

	50	Previous works with differences from the current study	☑	☐	☐	12-13
	51	Practical implications	☑	☐	☐	14
	52	Strengths and limitations (e.g., bias and generalizability issues)	☑	☐	☐	14
Open Science						
Data availability	53	Sharing images along with segmentation data [n/e]	☐	☑	☐	
	54	Sharing radiomic feature data	☑	☐	☐	
Code availability	55	Sharing pre-processing scripts or settings	☑	☐	☐	
	56	Sharing source code for modeling	☑	☐	☐	
Model availability	57	Sharing final model files	☐	☑	☐	
	58	Sharing a ready-to-use system [n/e]	☐	☐	☑	

Yes, details provided; **No**, details not provided; **n/e**, not essential; **n/a**, not applicable

Note: Use the checklist in conjunction with the main text for clarification of all items. Fill the "Page" column with the related page number where the information is provided.

Table S2 Details of breast MRI parameters.

Parameters	T1WI	T2WI	DWI	DISCO-DCE
Scan plane	Axial	Axial	Axial	Axial
Imaging technique	2D FSE	2D FSE	EPI	3D DISCO
Echo time(ms)	6.3	84.9	65.5	1.7
Repetition time(ms)	498	4975	2535	4.9
Fat suppression	NO	Yes	YES	YES
Field of view(mm)	360×360	360×360	360×360	360×360
Matrix	320×256	320×256	128×160	256×256
Section thickness(mm)	5	5	5	1.4
No. of sections	25	25	25	120/phase
Acceleration factors	2.5	2.5	2	2
Acquisition time(min:s)	1:40	1:59	2:32	19.4s/phase

Table S3 Features of differentiating HER2-enriched using phase and region one-to-one model

Model	Feature	Coefficient	OR
E_Intra			
	firstorder_10Percentile	0.1823	1.1999
	firstorder_TotalEnergy	0.3971	1.4875
	glcm_MCC	-0.2765	0.7584
	gldm_GrayLevelNonUniformity	-0.3923	0.6755
	glszm_LargeAreaHighGrayLevelEmphasis	0.2383	1.2691
	glszm_SmallAreaLowGrayLevelEmphasis	-0.1629	0.8497
	glszm_ZoneVariance	-0.2055	0.8142
	ngtdm_Contrast	-0.3783	0.6850
	ngtdm_Strength	-0.2774	0.7578
	shape_Elongation	-0.3455	0.7079
	constant	-0.9573	
E_Peri			
	firstorder_Kurtosis	0.2815	1.3251
	glcm_ClusterProminence	0.4624	1.5879
	glcm_Contrast	-0.3848	0.6806
	glcm_Idn	0.2539	1.2890
	gldm_LargeDependenceHighGrayLevelEmphasis	0.8183	2.2666
	gldm_LargeDependenceLowGrayLevelEmphasis	0.5137	1.6715
	glszm_GrayLevelNonUniformity	0.3661	1.4421
	ngtdm_Contrast	0.2889	1.3350
	constant	-0.9704	
P_Intra			
	firstorder_90Percentile	-0.2619	0.7696
	firstorder_Kurtosis	0.2798	1.3229
	glcm_ClusterProminence	-0.0337	0.9668
	glcm_ClusterShade	0.1715	1.1871
	glcm_Idmn	-0.3102	0.7333
	gldm_LargeDependenceHighGrayLevelEmphasis	0.4001	1.4920
	glszm_GrayLevelNonUniformity	-0.3276	0.7207
	glszm_SizeZoneNonUniformity	0.2482	1.2817
	glszm_ZoneVariance	-0.2796	0.7561
	ngtdm_Coarseness	-0.1728	0.8413
	ngtdm_Strength	-0.3359	0.7147
	shape_Elongation	0.1951	1.2154
	constant	-0.8804	
P_peri			
	firstorder_InterquartileRange	0.4975	1.6446
	firstorder_Kurtosis	0.2903	1.3368
	firstorder_Uniformity	1.1597	3.1890
	glcm_ClusterProminence	0.2125	1.2368

	glcm_MCC	0.2554	1.2910
	gldm_DependenceVariance	-0.5282	0.5897
	gldm_LargeDependenceLowGrayLevelEmphasis	0.0781	1.0812
	glrlm_LongRunLowGrayLevelEmphasis	0.0890	1.0931
	glrlm_RunLengthNonUniformity	0.2010	1.2226
	glszm_LargeAreaHighGrayLevelEmphasis	0.7375	2.0907
	ngtdm_Strength	0.1719	1.1876
	shape_MajorAxisLength	0.5402	1.7164
	constant	-1.009	
D_Intra			
	firstorder_Median	0.3037	1.3549
	glcm_ClusterShade	0.6846	1.9830
	gldm_LargeDependenceHighGrayLevelEmphasis	0.6957	2.0051
	gldm_LargeDependenceLowGrayLevelEmphasis	-0.4060	0.6663
	glszm_ZoneVariance	0.3058	1.3577
	ngtdm_Busyness	-0.0045	0.9955
	shape_Elongation	-0.3334	0.7165
	shape_Sphericity	-0.1014	0.9036
	constant	-1.0412	
D_Peri			
	firstorder_10Percentile	0.4214	1.5241
	firstorder_Kurtosis	0.4457	1.5616
	glcm_ClusterShade	0.0352	1.0358
	glcm_Idmn	-0.5147	0.5977
	glcm_MCC	0.6901	1.9939
	glrlm_RunLengthNonUniformity	0.3523	1.4223
	ngtdm_Strength	-0.3959	0.6731
	shape_Elongation	-0.1804	0.8349
	shape_SurfaceVolumeRatio	-0.2053	0.8144
	constant	-0.9371	

Table S4 Features of differentiating TNBC using phase and region one-to-one model

Model	Feature	Coefficient	OR
E_Intra			
	firstorder_10Percentile	-0.1658	0.8472
	firstorder_Kurtosis	0.2645	1.3028
	firstorder_TotalEnergy	-0.3993	0.6708
	glcm_Correlation	0.0770	1.0800
	glszm_SmallAreaLowGrayLevelEmphasis	0.4351	1.5451
	ngtdm_Busyness	-0.0891	0.9148
	ngtdm_Strength	0.2352	1.2651
	shape_Elongation	-0.3199	0.7262
	shape_SurfaceVolumeRatio	-0.7837	0.4567
	constant	-0.7851	
E_Peri			
	firstorder_Kurtosis	-0.2431	0.7842
	glcm_ClusterShade	-0.1028	0.9023
	glcm_Correlation	-0.7040	0.4946
	glcm_Idn	0.2567	1.2927
	gldm_LargeDependenceLowGrayLevelEmphas	-0.4376	
	is		0.6456
	glszm_GrayLevelNonUniformity	0.0507	1.0520
	constant	-0.7686	
P_Intra			
	firstorder_90Percentile	-0.1557	0.8558
	glcm_ClusterShade	-0.3027	0.7388
	gldm_LargeDependenceHighGrayLevelEmpha		
	sis	-0.1549	0.8565
	glszm_GrayLevelNonUniformity	-0.1029	0.9022
	glszm_ZoneVariance	0.1418	1.1523
	ngtdm_Coarseness	0.2162	1.2414
	shape_Elongation	-0.1958	0.8222
	constant	-0.7328	
P_Peri			
	firstorder_InterquartileRange	-0.0232	0.9771
	firstorder_Kurtosis	0.2870	1.3324
	glcm_MCC	-0.1000	0.9048
	gldm_DependenceVariance	-0.1752	0.8393
	gldm_LargeDependenceLowGrayLevelEmphas	-0.1492	
	is		0.8614
	glrlm_RunLengthNonUniformity	0.4050	1.4993
	ngtdm_Complexity	-0.9048	0.4046
	ngtdm_Strength	-0.0873	0.9164
	constant	-0.8037	
D_Intra			

	firstorder_Median	-0.3170	0.7283
	glcm_ClusterShade	-0.3678	0.6923
	glcm_Idn	0.9293	2.5327
	gldm_LargeDependenceHighGrayLevelEmphasis	-1.3391	0.2621
	gldm_LargeDependenceLowGrayLevelEmphasis	-0.2330	0.7922
	glszm_ZoneVariance	-0.5001	0.6065
	ngtdm_Coarseness	-0.3308	0.7183
	shape_Elongation	-0.4050	0.6670
	shape_Sphericity	0.6892	1.9921
	constant	-0.8883	
D_Peri			
	firstorder_10Percentile	-0.2820	0.7543
	firstorder_Kurtosis	-0.4045	0.6673
	glcm_ClusterShade	-0.0255	0.9748
	glcm_Idmn	0.4912	1.6343
	glcm_MCC	-0.5002	0.6064
	glrlm_RunLengthNonUniformity	-0.2605	0.7707
	shape_Elongation	-0.2851	0.7519
	shape_SurfaceVolumeRatio	-0.2059	0.8139
	constant	-0.7546	

Table S5 Features of differentiating Luminal using phase and region one-to-one model

Model	Feature	Coefficient	OR
E_Intra			
	firstorder_Kurtosis	-0.2422	0.7849
	firstorder_TotalEnergy	-0.1105	0.8954
	glszm_LargeAreaHighGrayLevelEmphasis	-0.1693	0.8443
	ngtdm_Contrast	0.2035	1.2257
	shape_Elongation	0.4187	1.5120
	constant	-0.6408	
E_Peri			
	firstorder_Kurtosis	-0.0993	0.905471
	glcm_ClusterProminence	-0.4075	0.665311
	glcm_ClusterShade	0.3281	1.388328
	glcm_Contrast	0.0196	1.019793
	glcm_Correlation	0.3964	1.486464
	glcm_Idn	-0.4923	0.611219
	gldm_LargeDependenceLowGrayLevelEmphasis	0.2570	1.293045
	glszm_GrayLevelNonUniformity	-0.4722	0.623629
	ngtdm_Contrast	-0.3732	0.688528
	constant	-0.6526	
P_Intra			
	firstorder_90Percentile	0.3961	1.4860
	firstorder_Kurtosis	-0.3954	0.6734
	glcm_ClusterProminence	-0.2234	0.7998
	glcm_ClusterShade	0.0749	1.0778
	glcm_Idmn	0.4003	1.4923
	gldm_LargeDependenceHighGrayLevelEmphasis	-0.1895	0.8274
	glszm_GrayLevelNonUniformity	0.3369	1.4006
	glszm_SizeZoneNonUniformity	-0.2105	0.8102
	glszm_ZoneVariance	0.0492	1.050
	ngtdm_Coarseness	-0.2275	0.7965
	ngtdm_Strength	0.5341	1.7059
	shape_Elongation	-0.0113	0.9888
	constant	-0.6125	
P_Peri			
	firstorder_InterquartileRange	-0.4174	0.6588
	firstorder_Kurtosis	-0.8013	0.4487
	glcm_MCC	-0.1655	0.8475
	gldm_DependenceVariance	0.3931	1.4816
	gldm_LargeDependenceLowGrayLevelEmphasis	0.1520	1.1642
	glrlm_RunLengthNonUniformity	-0.5437	0.5806

	ngtdm_Complexity	0.5616	1.7535
	ngtdm_Strength	0.1343	1.1437
	constant	-0.6432	
D_Intra			
	firstorder_Median	0.1113	1.1177
	glcm_ClusterShade	-0.2940	0.7453
	glcm_Idn	-0.5742	0.5632
	gldm_LargeDependenceHighGrayLevelEmphasis	0.1295	1.1383
	gldm_LargeDependenceLowGrayLevelEmphasis	0.2159	1.2410
	ngtdm_Busyness	-0.1417	0.8679
	ngtdm_Coarseness	0.2312	1.2601
	shape_Elongation	0.6261	1.8703
	shape_Sphericity	-0.4288	0.6513
	constant	-0.6600	
D_Peri			
	firstorder_Kurtosis	0.0751	1.0780
	firstorder_Range	0.0519	1.0533
	glcm_ClusterShade	-0.1027	0.9024
	glcm_Idmn	-0.0772	0.9257
	glcm_MCC	-0.1021	0.9029
	gldm_LargeDependenceLowGrayLevelEmphasis	0.3380	1.4021
	ngtdm_Strength	0.3839	1.4680
	shape_Elongation	0.4526	1.5724
	shape_SurfaceVolumeRatio	0.4161	1.5160
	constant	-0.6445	

Table S6 Features of differentiating Luminal A and Luminal B using phase and region one-to-one model

Model	Feature	Coefficient	OR
E_Intra			
	firstorder_Energy	0.4161	1.5160
	firstorder_TotalEnergy	0.3380	1.4021
	firstorder Variance	-0.3609	0.6971
	glcm ClusterProminence	0.2035	1.2257
	glcm_ClusterShade	-0.4412	0.6431
	gldm_HighGrayLevelEmphasis	0.4426	1.5567
	glrlm_LongRunHighGrayLevelEmphasis	-0.0993	0.9056
	glszm_LargeAreaHighGrayLevelEmphasis	0.1352	1.1448
	ngtdm_Complexity	0.3281	1.3883
	glcm_ClusterProminence	-0.341	0.7111
	constant	-0.5964	
E_Peri			
	firstorder_Energy	-0.5437	0.5806
	firstorder_TotalEnergy	-0.0751	0.9276
	firstorder_Variance	0.2641	1.3023
	glcm_ClusterProminence	0.0196	1.0197
	glcm_ClusterShade	-0.6374	0.5287
	gldm_LargeDependenceHighGrayLevelEmphasis	-0.4923	0.6112
	glrlm_RunLengthNonUniformity	0.2570	1.2930
	glszm_HighGrayLevelZoneEmphasis	0.4526	1.5724
	glszm_LargeAreaHighGrayLevelEmphasis	-0.3732	0.6885
	ngtdm_Complexity	-0.1105	0.8954
	constant	-0.6401	
P_Intra			
	firstorder_Energy	0.3961	1.4860
	firstorder_TotalEnergy	-0.3954	0.6734
	firstorder_Variance	-0.2234	0.7998
	glcm_Autocorrelation	0.0751	1.0780
	glcm_ClusterProminence	0.4003	1.4923
	glcm_ClusterShade	-0.1895	0.8274
	gldm_LargeDependenceHighGrayLevelEmphasis	0.3369	1.4006
	glrlm_LongRunHighGrayLevelEmphasis	-0.2105	0.8102
	glszm_LargeAreaHighGrayLevelEmphasis	0.0492	1.050
	ngtdm_Complexity	-0.2275	0.7965
	constant	-0.6712	
P_Peri			
	firstorder_10Percentile	-0.4174	0.6588
	firstorder_Kurtosis	0.0519	1.0533

	firstorder_Maximum	-0.1655	0.8475
	glcm_DifferenceAverage	0.3931	1.4816
	glrlm_RunLengthNonUniformity	0.1520	1.1642
	ngtdm_Strength	-0.1417	0.8679
	shape_LeastAxisLength	0.5616	1.7535
	shape_Maximum2DDiameterRow	0.1343	1.1437
	shape_MinorAxisLength	-0.1027	0.9024
	shape_VoxelVolume	-0.2422	0.7849
	constant	0.5794	
D_Intra			
	firstorder_TotalEnergy	-0.2940	0.7453
	firstorder_Variance	-0.5742	0.5632
	glcm_ClusterProminence	0.1295	1.1383
	glcm_ClusterShade	0.2159	1.2410
	gldm_HighGrayLevelEmphasis	-0.1693	0.8443
	glrlm_LongRunHighGrayLevelEmphasis	0.6261	1.8703
	glszm_LargeAreaHighGrayLevelEmphasis	-0.4288	0.6513
	constant	-0.6633	
D_Peri			
	firstorder_Energy	-0.4722	0.623629
	firstorder_TotalEnergy	0.0749	1.0778
	firstorder_Variance	-0.8013	0.4487
	glcm_ClusterProminence	-0.0772	0.9257
	glcm_ClusterShade	-0.1021	0.9029
	gldm_LargeDependenceHighGrayLevelEmphasis	0.3964	1.486464
	glszm_LargeAreaHighGrayLevelEmphasis	0.3839	1.4680
	glszm_SmallAreaHighGrayLevelEmphasis	-0.4075	0.665311
	ngtdm_Complexity	0.4187	1.5120
	constant	-0.5637	

Table S7 Features of differentiating HER2-enriched using combination model

Model	Feature	Coefficient	OR
E_Intra+Peri			
	Intra_firstorder_10Percentile	-0.3901	0.6770
	Intra_glcm_ClusterShade	0.1158	1.1228
	Intra_glcm_MCC	-0.5893	0.5547
	Intra_glrlm_ShortRunLowGrayLevelEmphasis	-0.2762	0.7587
	Intra_ngtdm_Contrast	0.0456	1.0467
	Intra_ngtdm_Strength	0.0595	1.0613
	Intra_shape_Elongation	-0.3235	0.7236
	Peri_firstorder_Kurtosis	0.2304	1.2591
	Peri_glcm_ClusterProminence	0.5221	1.6856
	Peri_glcm_Correlation	0.2223	1.2489
	Peri_glcm_Idn	0.4449	1.5603
	Peri_gldm_LargeDependenceHighGrayLevelEmphasis	0.6843	1.9824
	Peri_gldm_LargeDependenceLowGrayLevelEmphasis	0.3994	1.4909
	constant	-1.0757	
P_Intra+Peri			
	Intra_firstorder_90Percentile	-0.2852	0.7519
	Intra_firstorder_Kurtosis	0.3136	1.3683
	Intra_gldm_LargeDependenceHighGrayLevelEmphasis	0.2916	1.3386
	Intra_glszm_ZoneVariance	-0.4468	0.6397
	Intra_ngtdm_Coarseness	-0.0415	0.9593
	Intra_ngtdm_Strength	-0.5202	0.5944
	Intra_shape_Elongation	0.3927	1.4810
	Peri_firstorder_Kurtosis	0.4602	1.5844
	Peri_glcm_MCC	0.5196	1.6814
	Peri_gldm_DependenceVariance	-0.4812	0.6180
	Peri_glrlm_RunLengthNonUniformity	0.3074	1.3599
	Peri_ngtdm_Complexity	0.2886	1.3346
	constant	-1.0627	
D_Intra+Peri			
	Intra_glcm_ClusterShade	0.8662	2.3779
	Intra_gldm_LargeDependenceHighGrayLevelEmphasis	0.9724	2.6443
	Intra_gldm_LargeDependenceLowGrayLevelEmphasis	-1.5362	0.2152
	Intra_glrlm_GrayLevelNonUniformity	0.4076	1.5032
	Intra_glszm_ZoneVariance	0.6703	1.9548
	Intra_shape_Elongation	-0.6648	0.5144
	Intra_shape_Sphericity	0.1782	1.1951

Intra_shape_SurfaceVolumeRatio	1.2923	3.6412
Peri_firstorder_10Percentile	0.2683	1.3077
Peri_firstorder_Kurtosis	1.1906	3.2891
Peri_glcm_ClusterShade	-0.0536	0.9478
Peri_glcm_MCC	0.4211	1.5236
Peri_gldm_DependenceVariance	-0.7171	0.4882
Peri_ngtdm_Contrast	0.5135	1.6711
Peri_ngtdm_Strength	-0.2844	0.7525
Peri_shape_Elongation	0.5411	1.7179
constant	-1.2955	

Table S8 Features of differentiating TNBC using combination model

Model	Feature	Coefficient	OR
E_Intra+Peri			
	Intra_firstorder_Kurtosis	0.3576	1.4299
	Intra_glcm_MCC	0.2124	1.2366
	Intra_glrlm_ShortRunLowGrayLevelEmphasis	0.3340	1.3965
	Intra_glszm_ZoneVariance	0.0873	1.0912
	Intra_ngtdm_Busyness	-0.2520	0.7772
	Intra_shape_Elongation	-0.3820	0.6825
	Intra_shape_SurfaceVolumeRatio	-0.8066	0.4464
	Peri_glcm_ClusterProminence	-0.2612	0.7701
	Peri_glcm_ClusterShade	-0.5320	0.5874
	Peri_glcm_Correlation	-0.3097	0.7337
	Peri_gldm_LargeDependenceHighGrayLevelEmphasis	-0.8291	0.4364
	Peri_gldm_LargeDependenceLowGrayLevelEmphasis	-0.6869	0.5031
	Peri_glrlm_LongRunEmphasis	-0.5313	0.5878
	constant	-0.9274	
P_Intra+Peri			
	Intra_firstorder_90Percentile	-0.1405	0.8689
	Intra_glcm_ClusterProminence	0.1947	1.2149
	Intra_glcm_ClusterShade	-0.1805	0.8349
	Intra_gldm_LargeDependenceHighGrayLevelEmphasis	-0.2235	0.7997
	Intra_glszm_GrayLevelNonUniformity	-0.1102	0.8957
	Intra_glszm_ZoneVariance	0.1804	1.1977
	Intra_ngtdm_Coarseness	0.2020	1.2238
	Intra_shape_Elongation	-0.2027	0.8165
	Peri_firstorder_Kurtosis	0.1842	1.2023
	Peri_glcm_MCC	-0.1197	0.8872
	Peri_gldm_LargeDependenceLowGrayLevelEmphasis	-0.1710	0.8428
	Peri_glrlm_RunLengthNonUniformity	0.3130	1.3675
	Peri_ngtdm_Complexity	-0.7811	0.4579
	Peri_ngtdm_Strength	-0.1240	0.8834
	constant	-0.8336	
D_Intra+Peri			
	Intra_firstorder_Median	-0.1179	0.8888
	Intra_firstorder_Skewness	0.1500	1.1618
	Intra_glcm_ClusterShade	-0.1796	0.8356
	Intra_glcm_Correlation	0.6835	1.9808
	Intra_gldm_LargeDependenceHighGrayLevelEmphasis	-0.6390	0.5278

Peri_glcm_ClusterProminence	-0.1734	0.8408
Peri_glcm_MCC	-0.3371	0.7138
Peri_shape_Elongation	-0.2626	0.7690
constant	-0.8302	

Table S9 Features of differentiating Luminal using combination model

Model	Feature	Coefficient	OR
E_Intra+Peri			
	Intra_firstorder_Kurtosis	-0.2008	0.8181
	Intra_ngtdm_Contrast	0.2711	1.3114
	Intra_shape_Elongation	0.3927	1.4810
	Peri_firstorder_Skewness	-0.1368	0.8721
	Peri_glcm_Correlation	0.2426	1.2746
	Peri_glcm_Idn	-0.3692	0.6913
	Peri_glrlm_LongRunEmphasis	0.4426	1.5568
	constant	-0.6452	
P_Intra+Peri			
	Intra_firstorder_90Percentile	0.4097	1.5064
	Intra_firstorder_Kurtosis	-0.4321	0.6491
	Intra_glcm_ClusterProminence	-0.2064	0.8135
	Intra_glcm_ClusterShade	0.2012	1.2229
	Intra_glcm_Idmn	0.4085	1.5046
	Intra_gldm_LargeDependenceHighGrayLevelEmphasis	-0.2283	0.7959
	Intra_glszm_GrayLevelNonUniformity	0.3250	1.3840
	Intra_glszm_ZoneVariance	0.0720	1.0747
	Intra_ngtdm_Coarseness	-0.2688	0.7643
	Intra_ngtdm_Strength	0.6602	1.9352
	Intra_shape_Elongation	-0.0871	0.9166
	Intra_shape_SurfaceArea	-0.2648	0.7674
	Peri_firstorder_Kurtosis	-0.6125	0.5420
	Peri_glcm_MCC	-0.2724	0.7616
	Peri_gldm_DependenceVariance	0.3737	1.4531
	Peri_gldm_LargeDependenceLowGrayLevelEmphasis	0.1116	1.1181
	Peri_glrlm_RunLengthNonUniformity	-0.5157	0.5971
	Peri_ngtdm_Complexity	0.2737	1.3148
	Peri_ngtdm_Strength	0.0629	1.0649
	constant	-0.6792	
D_Intra+Peri			
	Intra_firstorder_Median	0.3380	1.4021
	Intra_glszm_GrayLevelVariance	0.8498	2.3392
	Intra_glszm_LargeAreaLowGrayLevelEmphasis	0.1197	1.1272
	Intra_shape_Elongation	0.4166	1.5168
	Intra_shape_SurfaceVolumeRatio	0.3620	1.4362
	Peri_glcm_ClusterShade	-0.3372	0.7138
	Peri_glcm_Idmn	-0.1972	0.8210
	Peri_glszm_GrayLevelNonUniformity	-0.0818	0.9215
	Peri_glszm_LargeAreaEmphasis	0.2358	1.2659

Peri_shape_Elongation	0.1751	1.1914
Peri_shape_SurfaceVolumeRatio	0.3588	1.4316
constant	-0.6849	

Table S10 Features of differentiating Luminal A and Luminal B using combination model

Model	Feature	Coefficient	OR
E_Intra+Peri			
	Intra_firstorder_Energy	-0.6125	0.5420
	Intra_firstorder_TotalEnergy	-0.2724	0.7616
	Intra_firstorder_Variance	0.3927	1.4810
	Intra_glcm_ClusterProminence	-0.1368	0.8721
	Intra_gldm_LargeDependenceHighGrayLevelEmphasis	0.0629	1.0649
	Intra_ngtdm_Complexity	-0.2008	0.8181
	Peri_firstorder_Energy	0.2426	1.2746
	Peri_firstorder_TotalEnergy	-0.3692	0.6913
	Peri_firstorder_Variance	0.1116	1.1181
	Peri_glcm_ClusterProminence	0.3250	1.3840
	constant	-0.5715	
P_Intra+Peri			
	Intra_firstorder_Energy	0.4097	1.5064
	Intra_firstorder_TotalEnergy	-0.4321	0.6491
	Intra_glcm_ClusterProminence	-0.2064	0.8135
	Intra_glcm_ClusterShade	0.2012	1.2229
	Peri_firstorder_Energy	0.4085	1.5046
	Peri_firstorder_TotalEnergy	-0.2283	0.7959
	Peri_firstorder_Variance	0.4426	1.5568
	Peri_glcm_ClusterProminence	0.0720	1.0747
	Peri_glcm_ClusterShade	-0.2688	0.7643
	Peri_ngtdm_Complexity	0.6602	1.9352
	constant	-0.5925	
D_Intra+Peri			
	Intra_firstorder_Energy	-0.0871	0.9166
	Intra_firstorder_TotalEnergy	-0.2648	0.7674
	Intra_glcm_ClusterProminence	0.1197	1.1272
	Intra_glcm_ClusterShade	0.4166	1.5168
	Intra_ngtdm_Complexity	0.2358	1.2659
	Peri_firstorder_Energy	-0.3372	0.7138
	Peri_firstorder_TotalEnergy	0.1751	1.1914
	Peri_firstorder_Variance	-0.5157	0.5971
	Peri_glcm_ClusterProminence	0.2737	1.3148
	Peri_glcm_ClusterShade	0.2711	1.3114
	constant	-0.7124	

Table S11 Selected features of full fusion model

Subtype	Feature	Coefficient	OR
Her2-enriched			
	E_Intra_firstorder_TotalEnergy	0.2818	1.3255
	E_Intra_glcm_Imc2	0.8530	2.3467
	E_Intra_gldm_GrayLevelNonUniformity	-0.5757	0.5623
	E_Intra_gldm_LargeDependenceHighGrayLevelEmphasis	0.2333	1.2628
	E_Intra_shape_Elongation	0.5766	1.7800
	E_Peri_glcm_Idmn	0.2589	1.2955
	P_Intra_glcm_Imc2	-1.0820	0.3389
	P_Intra_glcm_MCC	-0.3696	0.6910
	D_Intra_firstorder_Kurtosis	0.2374	1.2679
	D_Intra_firstorder_Median	-0.9813	0.3748
	D_Intra_glcm_MCC	-0.2879	0.7498
	D_Intra_gldm_LargeDependenceHighGrayLevelEmphasis	-0.3657	0.6937
	D_Intra_ngtdm_Contrast	-0.0128	0.9873
	D_Peri_firstorder_Range	-0.0066	0.9934
	D_Peri_glcm_MCC	-0.2028	0.8164
	D_Peri_glszm_ZoneVariance	1.0699	2.9151
	D_Peri_shape_SurfaceVolumeRatio	-0.4338	0.6480
	constant	-1.6439	
TNBC			
	E_Peri_firstorder_Kurtosis	0.1078	1.1138
	E_Intra_firstorder_Kurtosis	0.1278	1.1363
	E_Intra_glcm_DifferenceAverage	0.1076	1.1136
	E_Intra_glcm_MCC	0.0714	1.0740
	E_Intra_gldm_GrayLevelNonUniformity	0.1889	1.2079
	E_Intra_ngtdm_Busyness	-0.5459	0.5793
	P_Peri_glcm_MCC	-0.2156	0.8061
	P_Peri_gldm_LargeDependenceLowGrayLevelEmphasis	-0.1887	0.8280
	P_Peri_ngtdm_Strength	-0.3127	0.7315
	P_Intra_firstorder_90Percentile	-0.2284	0.7958
	P_Intra_glcm_Idmn	-0.2328	0.7923
	P_Intra_glszm_ZoneVariance	0.2696	1.3094
	P_Intra_shape_Elongation	-0.2489	0.7797
	D_Peri_shape_SurfaceVolumeRatio	-0.1395	0.8698
	D_Intra_firstorder_Median	-0.2543	0.7755
	D_Intra_glcm_ClusterShade	-0.3114	0.7324
	D_Intra_glcm_Idn	1.0267	2.7918
	D_Intra_gldm_LargeDependenceHighGrayLevelEmphasis	-1.5265	0.2173
	D_Intra_glszm_ZoneVariance	-0.3396	0.7121
	D_Intra_shape_Elongation	-0.0789	0.9241
	constant	-0.9511	
Luminal			

	E_Peri_firstorder_Kurtosis	-0.3807	0.6834
	E_Intra_firstorder_Kurtosis	-0.3402	0.7116
	E_Intra_gldm_GrayLevelNonUniformity	0.1427	1.1534
	E_Intra_glrlm_ShortRunLowGrayLevelEmphasis	-0.2621	0.7694
	E_Intra_ngtdm_Busyness	0.2260	1.2536
	P_Intra_firstorder_90Percentile	0.3734	1.4527
	P_Intra_firstorder_Kurtosis	-0.0314	0.9691
	D_Peri_shape_SurfaceVolumeRatio	0.3200	1.3771
	D_Intra_glcm_ClusterShade	-0.5134	0.5985
	D_Intra_glcm_Idn	-0.6141	0.5411
	D_Intra_gldm_LargeDependenceLowGrayLevelEmphasis	0.1613	1.1750
	D_Intra_glszm_ZoneVariance	-0.0430	0.9579
	D_Intra_shape_Elongation	0.4552	1.5765
	constant	-0.7062	
Luminal A vs. Luminal B			
	E_Intra_firstorder_Energy	-0.4509	0.6371
	E_Intra_firstorder_TotalEnergy	-0.3609	0.6970
	E_Intra_glcm_Imc1	0.1369	1.1467
	E_Peri_glcm_ClusterProminence	0.4236	1.5275
	P_Intra_glszm_LargeAreaHighGrayLevelEmphasis	-0.0695	0.9329
	D_Intra_shape_SurfaceVolumeRatio	0.3523	1.4223
	D_Intra_ngtdm_Strength	-0.6952	0.4990
	D_Peri_firstorder_Energy	0.1952	1.2156
	D_Peri_firstorder_TotalEnergy	0.5947	1.8125
	D_Peri_glcm_JointEnergy	-0.8310	0.4356
	constant	-0.6523	
